# Supplementary material for: Differential Effects of Whole Red Raspberry Polyphenols and Their Gut Metabolite Urolithin A on Neuroinflammation in BV-2 Microglia
Source: Int J Environ Res Public Health. 2020 Dec 24;18(1):68. doi: 10.3390/ijerph18010068 (PMC7795536; doi:10.3390/ijerph18010068)
Supplement: Supplementary file 1 [file ijerph-18-00068-s001.pdf]

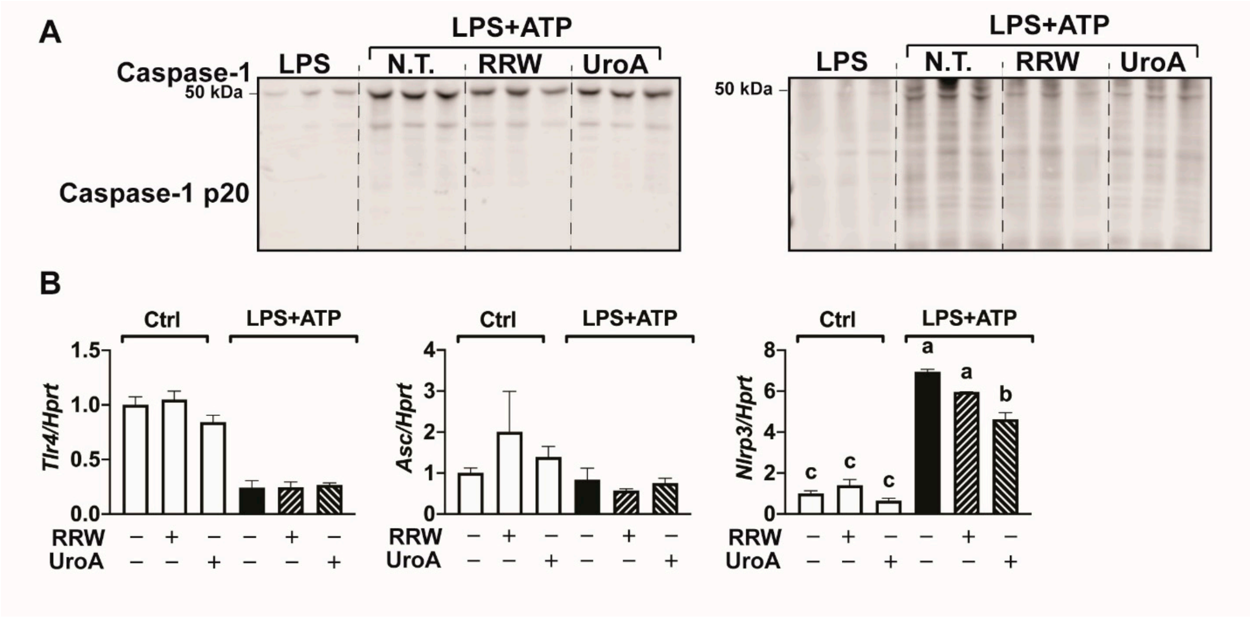

Figure S1. LPS with ATP treatment of BV-2 cells did not promote an inflammasome response. BV-2 cells treated with LPS (500 ng/mL) for 3 hours followed by ATP (2.5 mM) for 30 min with or without whole red raspberry polyphenols (10 µg/mL) and urolithin A (10 µM). (**A**) Protein expression of total supernatants from 6 well plates for caspase-1 and cleaved caspase-1 p20 and (right) total protein stained on the membrane. (**B**) Gene expression levels measured by RT-qPCR for *Asc*, *Tlr4*, and *Nlrp3*. All values are represented as the mean ± SEM. Treatments with different letters indicate significant differences by one-way ANOVA, $p < 0.05$. RRW = whole red raspberry polyphenols; UroA = urolithin A.

Table S1. Phenolic composition of whole red raspberry extracts.

Phenolic (mg per g Dry Extract)	Whole Red Raspberry (RRW)
Quercetin	2.73 ± 0.02
Myricetin	19.1 ± 0.02
Ellagic acid	66.1 ± 3.92
(+)-Catechin	11.4 ± 1.44
(−)-Epicatechin	75.4 ± 0.31
Anthocyanin	
Cyanidin 3-O-β-d-glucoside	8.73 ± 0.01
Cyanidin 3-O-β-d-glucoside equivalent	39.6 ± 0.04
Total phenolic content (mg of GAE per g)	279.0 ± 31

Values represent the mean ± SD of triplicate tests. Ellagitannin is calculated as ellagic acid after hydrolysis. The Cyanidin-3-sophoroside peak is quantified as Cyanidin 3-*O*-d-glucoside equivalent. GAE, gallic acid equivalent. Adapted from Rong et al., 2020.

Table S2. Primer sequences for qPCR.

Gene	Forward/Reverse	Sequence (5′-3′)
Il1b	Forward	TCCAGGATGAGGACATGAGCAC
	Reverse	GAACGTCACACACCAGCA GGTTA
Il6	Forward	CCCCAATTTCCAATGCTCTCCT
	Reverse	CGCACTAGGTTTGCCGAGTA

Tnf	Forward	AACTTCGGGGTGATCGGTCC
	Reverse	GGTGGTTTGTGAGTGTGAGGG
iNos	Forward	TTGGTGAAGGGACTGAGCTGT
	Reverse	CCGTGGAGTGAACAAGACCCA
Ym1	Forward	AGACTTGCGTGACTATGAAGCATTG
	Reverse	GCAGGTCCAAACTTCCATCCTC
Nlrp3	Forward	ATGCTGCTTCGACATCTCCT
	Reverse	AACCAATGCGAGATCCTGAC
Tlr4	Forward	AGTGGGTCAAGGAACAGAAGCA
	Reverse	CTTTACCAGCTCATTTCTCACC
Asc	Forward	ATGCCATCCTGGACGCTCTT
	Reverse	ATGCGCCCATAGCCTTCTCG
Hprt	Forward	TTGCTCGAGATGTCATGAAGGA
	Reverse	AGCAGGTCAGCAAAGAACTTATAGC

Table S3. List of antibodies used in Western blotting.

Antibody Name	Catalog Number	Company	Isotype	Dilution
P-JNK (Thr183/Tyr185)	4668	Cell Signaling Technology	Rabbit	1:1000
T-JNK (SAP/JNK)	9252	Cell Signaling Technology	Rabbit	1:1000
p-c-Jun (Ser73)	3270	Cell Signaling Technology	Rabbit	1:1000
c-Jun	9165	Cell Signaling Technology	Rabbit	1:1000
Anti-caspase-1 (p20)	AG-20B-0042-C100	Adipogen Life Sciences	Mouse	1:500
